# Editorial: Rising stars in fungal pathogenesis: 2023

**DOI:** 10.3389/fcimb.2024.1484527

**Published:** 2024-09-30

**Authors:** Brian L. Wickes, Adriana Marcela Celis Ramírez, Michal A. Olszewski, Yong-Sun Bahn

**Affiliations:** ^1^ Department of Microbiology, Immunology, and Molecular Genetics, University of Texas Health Science Center at San Antonio, San Antonio, TX, United States; ^2^ Departamento Ciencias Biológicas, Facultad de Ciencias, Universidad de los Andes, Bogotá, Colombia; ^3^ Microbiology & Immunology and Pulmonary & Critical Care Medicine, University of Michigan Medical School, Ann Arbor, MI, United States; ^4^ Research Service, Department of Veterans Affairs Medical Center, Ann Arbor, MI, United States; ^5^ Department of Biotechnology, College of Life Science and Biotechnology, Yonsei University, Seoul, Republic of Korea

**Keywords:** *Cryptococcus neoformans*, *Candida albicans*, *Candida auris*, fungal pathogenesis, rising stars

In recent decades, invasive fungal infections and diseases have emerged as a significant public health treat, affecting millions of people worldwide each year with unacceptably high mortality rates (Denning, 2024). To exacerbate the situation, the repeated use of a limited range of antifungal agents in both clinical and environmental settings has led to the rise of multidrug-resistant fungal strains, making it increasingly challenging to control fungal infections (Fisher et al., 2022). This situation underscores the urgent need for a thorough understanding of fungal pathogenesis mechanisms and the identification of new targets for antifungal drug development. It also calls for a new generation of investigators to address these critical issues through their research. This Research Topic aims to showcase the work of “Rising Stars”, early to mid-career researchers focused on the pathobiological characteristics of *Candida albicans*, *Candida auris*, and *Cryptococcus neoformans*—all recently classified as critical fungal pathogens by the World Health Organization (WHO).

Kwon et al.‘s group at the Korean Atomic Energy Research Institute (KAERI) has characterized the pathobiological roles of the LAMMER kinase, Lkh1, in *Cryptococcus neoformans* (Kwon et al.), a major fungal pathogen responsible for over 140,000 deaths annually due to meningoencephalitis (Denning, 2024). Since joining KAERI, Dr. Jung has focused on elucidating the DNA damage response and repair mechanisms in *C. neoformans*, which is notably resistant to radiation (Jung et al., 2017). In their current study (Kwon et al.), they demonstrated that Lkh1 plays a crucial role in DNA damage response through both Rad53-dependent and independent pathways, and its deletion completely abolishes the virulence of this basidiomycetous pathogen, reflecting its pleiotropic pathobiological roles. Although LAMMER kinases are evolutionarily conserved from fungi to humans, which poses challenges for developing antifungal drugs targeting them, future comparative structural analyses of fungal and human LAMMER kinases may open avenues for designing specific inhibitors that could serve as potent antifungal drug candidates.

Goughenour et al. is a recently appointed Assistant Professor in the Departments of Internal Medicine and Microbiology & Immunology at the University of Michigan Medicine. She is also a new investigator at the Ann Arbor VA Medical Center holding a career development award. Her research focuses on host-pathogen interactions in the context of cryptococcal infections, investigating the cryptococcal genes required for virulence (Stempinski et al., 2022) and the role of host factors such as inducible nitric oxide synthase (iNOS) in providing protection (Goughenour et al., 2021). In this Research Topic, her studies demonstrate how the cryptococcal virulence factor Trehalose-6-phosphate synthase (Tps1) affects host-fungal interactions across multiple layers of host defenses (Goughenour et al.). Tps1 represents a promising antifungal target because this pathway is not conserved in vertebrates. The study reveals that intercepting this pathway rendered the fungus avirulent in animal models. The critical role of Tps1 in *C. neoformans’* evasion of pulmonary and innate defense mechanisms was demonstrated and linked to Tps1’s involvement in cryptococcal capsule formation. Thus, novel therapeutics that inhibit Tps1 could reduce both fungal fitness and its resistance to host defense mechanisms.

Brandquist et al.‘s group at the University of Nebraska has focused on characterizing filamentation phenotypes in clinical *C. albicans* strains. Brandquist et al. has been extensively researching various pathobiological aspects of *C. albicans*, including serum sensitivity, biofilm formation, cell wall regulation, iron regulation, and dimorphic switching. Among these, filamentation is the most well-known virulence factor of *C. albicans*. In their current study (Brandquist et al.), the team genotyped 20 clinical *C. albicans* strains collected from the Nebraska Medicine clinical laboratory using multilocus sequence typing (MLST) analysis. Interestingly, these strains proved to be as diverse as those collected from across the United States. The study’s most notable finding is that all these clinical *C. albicans* strains exhibited robust filamentation in liquid-inducing media but not in solid-inducing media, while the standard wild-type strain SC5314 filaments well in both conditions. Consequently, the authors suggest that traditional solid filamentation assays may not be reliable models for assessing the filamentation efficiency of diverse *C. albicans* isolates and may not be suitable for drug development.

Vélez et al., part of the Studies in Translational Microbiology and Emerging Diseases (MICROS) Research Group, has evaluated the antifungal activity of the C14R peptide against a large set of clinical isolates of *C. albicans* and *C. auris* (Vélez et al.) — pathogens that cause prevalent and life-threatening invasive fungal infections worldwide, often associated with azole resistance and therapeutic failure (McCarty et al., 2021). This study found that the C14R peptide demonstrated strong antifungal activity and exhibited a synergistic effect with fluconazole, even in isolates resistant to this azole (Vélez et al.). The researchers suggest that this combination could potentially enhance therapeutic efficacy and reduce the required doses of this antifungal. Additionally, C14R was shown to decrease biofilm biomass, reduce cell viability, and induce morphological and structural alterations in the yeasts. These findings provide promising evidence that could contribute to combating antifungal resistance.

Luther et al.‘s team at the University of Würzburg used a systems biology approach that combined multiple datasets with experimental data to tease out the regulatory functions of two serine-arginine protein kinases, Sky1 and Sky2, in *Candida albicans*. A previous study of these proteins revealed that Sky1 is involved in the regulation of ion homeostasis and Sky2 is important for dipeptide utilization (Brandt et al., 2022). Luther et al.‘s team generated RNA-seq profiles of *sky1Δ* and *sky2Δ* mutants and then integrated these profiles with phosphoproteome data. They then developed information on protein-protein interactions to yield information linking signaling proteins and their transcription factors to produce signaling modules, which ultimately enabled a new understanding of signaling cascades and transcriptional regulation. A deep analysis of kinase signaling networks connected SKY1 and SKY2 to morphology, stress and chemical resistance, all of which play important roles in *Candida albicans* virulence. This study demonstrates the power of applying a systems biology approach, common in mammalian systems, to human fungal pathogens as it provides a novel strategy for dissecting the transcriptional regulation of complex signaling cascades.

The research highlighted in this Research Topic underscores the urgent need for innovative approaches to address the growing threat of invasive fungal infections. From uncovering the molecular mechanisms of fungal virulence to identifying potential antifungal targets and evaluating novel therapeutic strategies, these studies collectively contribute to advancing our understanding of fungal pathogenesis. As multidrug-resistant strains continue to emerge, the work of these “Rising Stars” showcased here is pivotal in paving the way for developing effective antifungal therapies and the broader fight against fungal diseases.
